# An update on the use of image-derived input functions for human PET studies: new hopes or old illusions?

**DOI:** 10.1186/s13550-023-01050-w

**Published:** 2023-11-10

**Authors:** Tommaso Volpi, Lucia Maccioni, Maria Colpo, Giulia Debiasi, Amedeo Capotosti, Tommaso Ciceri, Richard E. Carson, Christine DeLorenzo, Andreas Hahn, Gitte Moos Knudsen, Adriaan A. Lammertsma, Julie C. Price, Vesna Sossi, Guobao Wang, Paolo Zanotti-Fregonara, Alessandra Bertoldo, Mattia Veronese

**Affiliations:** 1 Department of Radiology and Biomedical Imaging, Yale University, 801 Howard Avenue, PO Box 208048, New Haven, CT 06520-8048 USA; 2 Department of Information Engineering, University of Padova, Padua, Italy; 3 Padova Neuroscience Center, University of Padova, Padua, Italy; 4 Department of Surgery, Oncology and Gastroenterology, University of Padova, Padua, Italy; 5 Fondazione Policlinico Universitario A. Gemelli IRCCS, Rome, Italy; 6 Neuroimaging Laboratory, Scientific Institute IRCCS Eugenio Medea, Bosisio Parini, LC Italy; 7 Renaissance School of Medicine, Stony Brook University, Stony Brook, NY USA; 8 Department of Psychiatry and Psychotherapy, Comprehensive Center for Clinical Neurosciences and Mental Healthy (C3NMH), Medical University of Vienna, Vienna, Austria; 9 Neurobiology Research Unit, Copenhagen University Hospital Rigshospitalet, Copenhagen, Denmark; 10 Department of Clinical Medicine, University of Copenhagen, Copenhagen, Denmark; 11 Department of Nuclear Medicine and Molecular Imaging, University of Groningen, Groningen, Netherlands; 12 Athinoula A. Martinos Center for Biomedical Imaging, Massachusetts General Hospital, Boston, USA; 13 Department of Physics and Astronomy, University of British Columbia, Vancouver, BC Canada; 14 Department of Radiology, University of California Davis Medical Center, Sacramento, CA USA; 15 Molecular Imaging Branch, National Institute of Mental Health, Bethesda, MD USA; 16 Department of Neuroimaging, King’s College London, London, UK

**Keywords:** Image-derived input function, Blood sampling, Long axial field of view, High sensitivity, Total-body PET

## Abstract

**Background:**

The need for arterial blood data in quantitative PET research limits the wider usability of this imaging method in clinical research settings. Image-derived input function (IDIF) approaches have been proposed as a cost-effective and non-invasive alternative to gold-standard arterial sampling. However, this approach comes with its own limitations—partial volume effects and radiometabolite correction among the most important—and varying rates of success, and the use of IDIF for brain PET has been particularly troublesome.

**Main body:**

This paper summarizes the limitations of IDIF methods for quantitative PET imaging and discusses some of the advances that may make IDIF extraction more reliable. The introduction of automated pipelines (both commercial and open-source) for clinical PET scanners is discussed as a way to improve the reliability of IDIF approaches and their utility for quantitative purposes. Survey data gathered from the PET community are then presented to understand whether the field’s opinion of the usefulness and validity of IDIF is improving. Finally, as the introduction of next-generation PET scanners with long axial fields of view, ultra-high sensitivity, and improved spatial and temporal resolution, has also brought IDIF methods back into the spotlight, a discussion of the possibilities offered by these state-of-the-art scanners—inclusion of large vessels, less partial volume in small vessels, better description of the full IDIF kinetics, whole-body modeling of radiometabolite production—is included, providing a pathway for future use of IDIF.

**Conclusion:**

Improvements in PET scanner technology and software for automated IDIF extraction may allow to solve some of the major limitations associated with IDIF, such as partial volume effects and poor temporal sampling, with the exciting potential for accurate estimation of single kinetic rates. Nevertheless, until individualized radiometabolite correction can be performed effectively, IDIF approaches remain confined at best to a few tracers.

**Supplementary Information:**

The online version contains supplementary material available at 10.1186/s13550-023-01050-w.

## Background

Positron emission tomography (PET) provides remarkable insight into human physiology and pathophysiology due to its ability to reveal interactions between radioligands and enzymes, receptors, and transporters.

While most clinical PET applications remain confined to single-frame, static imaging, research PET experiments typically require long dynamic acquisitions (60–120 min) to fully describe the tracer kinetics and allow its quantification by compartment modeling and related approaches. Common outcome parameters include single rate constants (*K*_1_ [mL/cm^3^/min]), *k*_2_ [min^−1^], *k*_3_ [min^−1^], *k*_4_ [min^−1^]), and/or macrokinetic parameters (e.g., net influx constant *K*_i_ [mL/cm^3^/min], total distribution volume *V*_T_ [ml/cm^3^], binding potential *BP*_ND_ [unitless]) [1]. These parameters are useful for many applications, such as the measurement of drug occupancy, or the assessment of new radioligands [2].

The gold-standard in vivo approach to kinetic parameter estimation often requires measuring the metabolite-corrected tracer concentration in arterial plasma (*C*_p_ [kBq/mL]), i.e., the arterial input function (AIF), which requires arterial sampling. Arterial cannulation is not particularly dangerous: Local complications arise only in rare cases and are usually resolved by medical intervention [3]. However, arterial sampling is still an invasive procedure which can cause discomfort, and may discourage participation in PET research studies [4], especially for elderly and fragile individuals, or when repeated cannulations are required. Moreover, blood sampling adds to the total cost of PET acquisition, due to the need for specialized equipment and personnel (e.g., anesthesiologist to insert the arterial line, laboratory personnel to handle blood samples and perform radiometabolite correction, etc.). Sampling errors can also occur, and the AIF needs to be “noise-free” to avoid error propagation to kinetic estimates [5].

To eliminate the invasiveness of arterial sampling, three of the most popular alternative approaches which have been proposed to potentially achieve absolute quantification of PET data are: (1) *image-derived input functions* (IDIF), i.e., obtaining the input from blood pools within the PET images themselves [3, 6], (2) *population-based input functions* (PBIF), i.e., using an average AIF from an independent subject group after rescaling it with individual information, such as one or two blood samples [7], (3) *simultaneous estimation* (SIME) of kinetic parameters and the input function itself from multiple tissue time-activity curves (TACs) obtained from PET images [8]. Specifically, as fully detailed in [3], PBIF approaches have many advantages (they are simple and independent of the scanner’s technical characteristics), but can suffer from misestimation of the peak and metabolite fraction, while SIME approaches incur into identifiability issues and thus still require some “anchors” (usually, one or more blood samples) for accurate estimation of the metabolite-corrected input. Other approaches for deriving the blood input non-invasively include the use of wrist positron detectors [9], or even dual-PET systems for scanning the heart (for input extraction) simultaneously with another organ, e.g., the brain [10]. In addition, if a reference region devoid of specific target binding (and with comparable blood-to-tissue exchanges to the other regions) is available, it can be used as a surrogate input [11]. However, it can be challenging to identify such a region in practice, and reference-tissue approaches lack the ability to determine the absolute value of some kinetic parameters, especially *K*_1_.

**Fig. 1:**
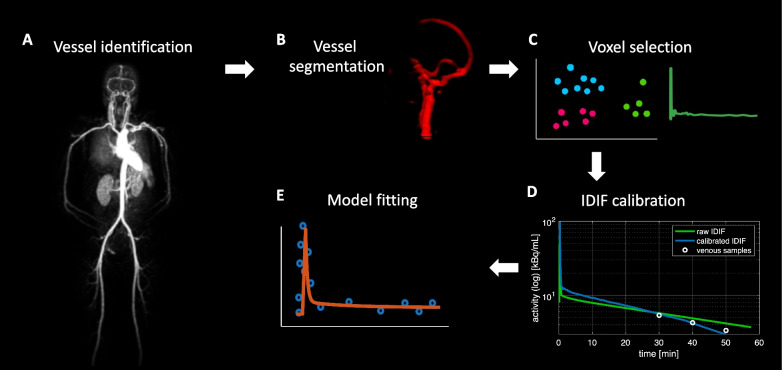
IDIF extraction pipeline. Example of a pipeline for IDIF extraction as typically adopted in the literature: vessel identification (**A**), vessel segmentation (**B**), voxel selection (**C**), partial volume effect correction (according to Chen’s approach [6]) (**D**), model fitting (**E**)

IDIF seems to provide a rather simple and attractive solution to eliminate the invasiveness of arterial sampling. This approach typically requires identification of a vascular structure in the PET field of view (FOV), segmentation or delineation of a region of interest (ROI) in the vessel to extract the TAC; correction for partial volume effects (PVEs) (Fig. 1); finally, corrections for plasma vs. blood differences and for radiometabolites are required (*see below*). In some cases, approaches to extract IDIF directly without segmentation have also been proposed, such as blind source separation [12] or machine/deep learning [13].

Despite its theoretical advantages, the actual usefulness of IDIF has been questioned, particular with regard to brain imaging [3], where IDIF is used in a minority of studies. This skepticism toward IDIF is due to a variety of factors: (1) the impact of PVEs in small vessels; (2) IDIF’s inability to account for radiometabolite activity and difference between plasma and whole-blood tracer concentrations; (3) its susceptibility to motion; and (4) its dependence on the settings of reconstruction algorithms (including the reconstruction time grid and smoothing level).

This paper provides a brief update of IDIF approaches in human PET studies. A strong focus is placed on advances made in the last decade and efforts to make IDIF more accurate and reproducible for conventional, limited axial FOV PET scanners; such efforts include the development of IDIF extraction pipelines in open-source and commercial software [14, 15].

The paper also presents results from a survey disseminated to the PET imaging community that sought to gather information about their experience implementing IDIF approaches and gauge current opinion on the use of IDIF, especially for brain PET [3].

Finally, the paper discusses how next-generation PET scanners are starting to provide interesting possibilities for overcoming current IDIF limitations, including the advantages of long axial field of view (LAFOV) scanners and scanners with ultra-high sensitivity and spatial and temporal resolution [16].

## Main text

### The known issues of IDIF approaches

#### Vessel size and partial volume effects

Among the most relevant factors determining the feasibility of an IDIF approach is the *vessel diameter*. When large vessels are available in the FOV, IDIF extraction has been successful since the early days of PET. The thoracic aorta (inner diameter: 2.5–3 cm) and the left ventricle have been frequently employed as extraction sites for studies of the heart [17] and lung [18]. Similarly, the abdominal aorta (~ 2 cm) has been used for liver imaging [19] and the common iliac arteries (~ 1 cm) for prostate cancer studies [20]. The availability of the aorta and other large blood pools in the FOV has made IDIF the prevailing approach for input function extraction in cardiac and oncological studies, so that IDIF-based quantification of myocardial blood flow (MBF) (using ^82^Rb, [^15^O]H_2_O and other perfusion tracers [21]) has been successfully brought to the clinic.

IDIF extraction is instead known to be very challenging when only small-diameter, convoluted vessels, and/or with complex surrounding anatomy, are present inside the FOV. Brain studies, in particular, have had to face this issue, with the internal carotid artery having a caliber of ~ 4 to 5 mm, which is smaller than the spatial resolution of most clinical PET scanners (~ 5 mm) [3]. The limited PET spatial resolution relative to the vessel diameter introduces *PVEs* and *spillover* effects (spill-out and spill-in), which lead to loss of signal for objects smaller than 2 times the scanner’s point spread function (PSF) full width at half maximum (FWHM) [22], and can alter the amplitude and shape of the vascular signal [3]. Even in the case of the HRRT, i.e., the brain-dedicated scanner with the highest sensitivity and spatial resolution until recently, extracting a carotid IDIF free of PVE artifacts was shown not to be feasible [23]. Of course, in case of vascular pathology (e.g., arterial inflammation or trauma), the situation is made even more complex by potentially increased tracer uptake in the vessel wall, which produces spill-in effects.

To try and minimize the artifacts affecting IDIFs obtained from difficult extraction sites, multiple correction and calibration approaches have been developed, involving the use of blood samples or the estimation of recovery coefficients from knowledge of the vessel volume and scanner resolution, as detailed in [3]. The methods requiring blood samples are typically expected to be the most accurate [3]. Using *venous* instead of arterial samples for calibration is sometimes possible, if arteriovenous equilibrium occurs within the scan duration, with the advantage of lower invasiveness [6]. Next-generation PET scanners with LAFOV, increased sensitivity and spatial resolution, seem to be very promising for this issue [16].

#### The unsolved issue of radiometabolite correction

Another main argument against IDIF approaches is that they usually still require blood sampling to account for *radiometabolite* activity. To obtain the proper input function (i.e., parent concentration in the arterial plasma), whole blood must first be centrifuged to separate the plasma, and the parent concentration in plasma must be separated from that of radiometabolites. With some notable exceptions, like [^18^F]fluorodeoxyglucose ([^18^F]FDG), the majority of PET tracers produce radiometabolites, which contribute to the measured radioactivity in the blood, and image-based approaches cannot distinguish them from the parent compound [3].

A population plasma parent fraction has been used, even in recent work, in case of low inter-individual variability [24]. For tracers with significant between-subject variability in the parent fraction (which can depend on various physiological or pathophysiological factors, e.g., sex, diseases, or drugs affecting hepatic function, etc. [3]), the use of a few late venous samples (when metabolite concentration is maximal) has been proposed [24, 25]. However, great caution is required because arterial and venous metabolite concentrations often differ, even at late time points [26]. Moreover, some authors have proposed using a reduced number of arterial blood samples, but of course this invalidates the non-invasiveness of the procedure [27].

Additionally, tracer concentrations in plasma and whole blood are often not the same and the *plasma-over-blood (POB) ratio* often varies over the duration of a PET scan [28]; in this case the use of venous samples, or a population-average curve scaled with venous samples, was shown to be an effective strategy for some tracers [24].

While the POB issue seems easier to address, the radiometabolite correction problem remains open. However, new solutions may come with the help of next-generation scanners.

#### The impact of motion and reconstruction

*Motion* artifacts are known to have a significant impact on long dynamic PET acquisitions [29], and their correction is important to improve IDIF accuracy, especially when calibration with blood samples is not performed [27]. In the thorax and abdomen, motion has a stronger impact, since, unlike head motion, body motion is non-rigid. However, motion artifacts have been remarkably under-explored in the non-brain PET literature [29, 30]. This is an important issue, as body motion can result in severe misplacements of the vessel ROIs required for IDIF extraction, e.g., aorta [15].

The impact of image *reconstruction* on IDIF methods has been less explored. The choice of the reconstruction method (filtered back-projection, FBP vs. ordered subset expectation maximization, OSEM) may affect the quality of the IDIF curve. Scatter correction, which has been recognized as critically important to avoid biased kinetic estimates [31], is expected to impact IDIF estimation as well [3], especially at late times when the vascular tracer concentration is very low, and thus more susceptible to scatter artifacts. Additionally, noise reduction can have an important impact on IDIF recovery: The appropriate adjustment of the smoothing parameters, during and after reconstruction, is crucial to avoid biased estimates [32, 33]; moreover, advanced denoising approaches added to reconstruction have been shown to improve the match between IDIF and blood samples for [^18^F]FDG [34]. A critical aspect of image reconstruction is also selecting the PET frame duration, which requires a compromise between describing the rapid variations of the early IDIF and maintaining a sufficiently high signal-to-noise ratio (SNR), which has been particularly difficult for low-sensitivity scanners [35]. Next-generation scanners are expected to lead to both new opportunities and new challenges in the area of motion correction and reconstruction.

For these reasons, especially in brain PET, IDIF approaches have failed to reach widespread applicability [3]. However, developments in scanner technology in the last decade—particularly the availability of next-generation PET scanners—may be able to address these critical issues, fostering a new era for IDIF and non-invasive quantitative PET.

### Automatic IDIF extraction: open-source and commercial pipelines for conventional PET scanners

Significant advances have been made during the last decade to make IDIF extraction more reproducible and reliable on conventional scanners with limited axial FOV (i.e., 15–30 cm). For instance, to obviate the variability of manual drawing of ROIs [36], automated pipelines have been developed [15, 37]. Some of these approaches have been included into fully automated routines for parametric imaging (i.e., voxel-wise mapping of the kinetic parameters of interest) (for an extensive list, see [14]).

*Cardiac* applications have already reached the clinic [21], thanks to the availability of large blood pools in the FOV. Specifically, multiple commercially or publicly available pipelines for quantification of MBF and MBF reserve have been developed, e.g., QPET [38], PMOD routines (http://www.pmod.com/), SyngoMBF (https://www.siemens-healthineers.com), Carimas [39], Cardiac VuEr [40], FlowQuant [41]. These pipelines are usually applicable to any cardiac perfusion tracer (i.e., ^82^Rb, [^15^O]H_2_O, [^13^N]ammonia, etc.). The software packages implement various strategies for IDIF extraction, e.g., (1) automated or semi-automated ROI placement, typically in the left ventricle, or the ascending aorta, (2) approaches based on factor analysis or clustering, or (3) hybrid approaches with ROI segmentation and factor analysis. Body and respiratory motion can potentially limit the accuracy of these methods [21, 29], as it can lead to misplacement of the ROI used for IDIF extraction, especially for longer stress studies; however, motion correction is not implemented in most of the mentioned software packages. Some studies that tested and compared the performance of these pipelines found good agreement between software packages [42, 43], but systematic evaluations of ^82^Rb studies on large cohorts reported significant differences in the estimates of MBF when comparing ROI-based methods with factor analysis [44]. Moreover, automated ROI placement was found to be unreliable for some of these pipelines (e.g., PMOD), with 30% failure rates, thus requiring manual adjustment [43].

Multibed–multipass imaging has made it feasible to perform *whole-body* dynamic PET imaging with conventional PET scanners [45], with important applications for e.g., oncology [46]. With a continuous bed motion approach, the first 5 min can be dedicated to a single-bed acquisition over the heart to capture the early IDIF kinetics, followed by multiple rapid whole-body passes to measure tissue kinetics and the IDIF tail [30]. Commercial software for whole-body parametric imaging, with a focus on [^18^F]FDG, has been made available by multiple vendors, including Siemens [15], GE, and United Imaging [47]. For instance, the *FlowMotion MultiParametric PET* suite, developed by Siemens for clinical PET/CT scanners with limited FOV (e.g., Biograph mCT, Vision 600 [48]), allows for IDIF extraction from the descending aorta or left ventricle, which are automatically identified on a low-dose computed tomography (CT) scan using the ALPHA machine learning algorithm; a ROI is placed and registered to PET images to extract the IDIF [15]. The ROI can be manually adjusted, if necessary. Although clear assessment criteria were missing, the success rate for this automated ROI placement was reported to be 95% [15], and it has already been applied in multiple studies, both with [^18^F]FDG [48, 49] and other tracers [50]. However, full comparison with the AIF showed that the automated ROI placement requires rigorous quality control, and performing additional motion correction is advisable [51]. It should also be noted that these whole-body acquisition routines require a compromise: if early scanning is dedicated to a chosen blood pool, full-compartmental analysis non-feasible [30].

**Fig. 2:**
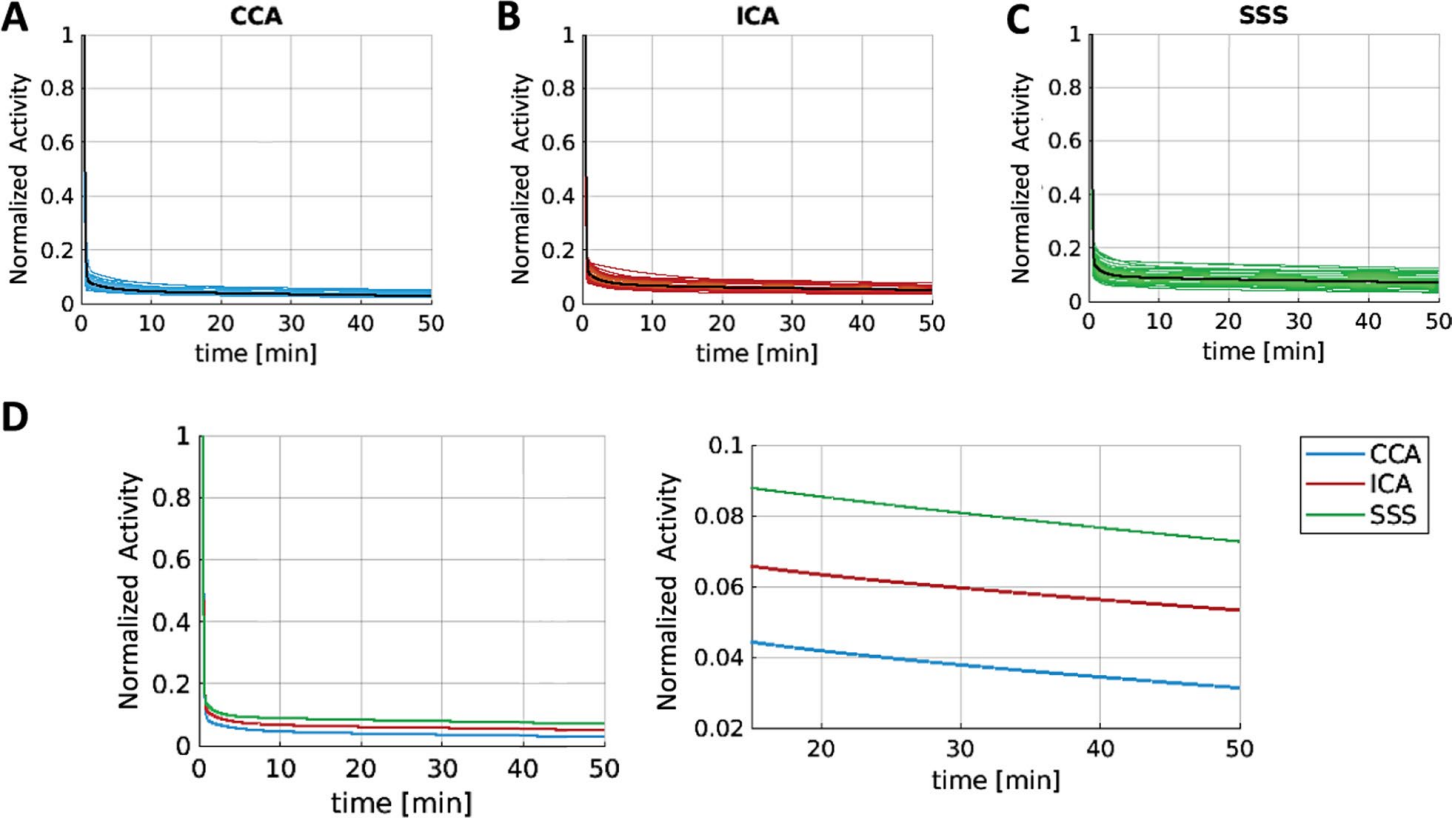
Comparing IDIF sites for brain PET. Comparison of three IDIF extraction sites (common carotid artery (CCA); internal carotid artery (ICA); superior sagittal sinus (SSS)) in 38 patients with glioma (brain PET acquisitions performed on a Biograph mMR scanner). IDIF curves (after the peak, fitted with a three-exponential decay model, and normalized by their maximum) shown at the individual (colored) and population mean (black) level for each extraction site (panel **A**: CCA, panel **B**: ICA, panel **C**: SSS). SSS had the highest between-subject variability, and CCA had the lowest. Panel **D** shows the mean fitted IDIFs (full-time course on the left, 20–50-min portion on the right). The curves are almost parallel, with CCA as the lowest and SSS the highest, thus suffering from highest spillover. As a note, the diameters of the three vessels are: CCA ~ 6 to 7 mm, ICA ~ 4 to 5 mm, SSS ~ 3 to 4 mm

Commercial software is not yet available for dedicated brain imaging, where the FOV does not typically include large vessels. However, a variety of open-source approaches have been developed, especially for *hybrid PET/MR scanners*. The first step is usually vessel segmentation, which can be performed either on anatomical images (MR, CT) or directly on early (perfusion-weighted) PET images to avoid coregistration issues [52, 53]. For PET/MRI, using time-of-flight MR angiography, in turn, can provide better vessel segmentation, thus minimizing coregistration issues [54]; moreover, concomitant MR acquisitions (for monitoring head motion) simplify PET motion correction [37, 46]; also, PET image quality may be significantly improved by using MR anatomical prior information during image reconstruction [16]. Due to a relatively longer axial FOV (e.g., 26 cm for Biograph mMR [55]), these scanners also allow imaging of larger vascular structures in the neck, including part of the common carotids (6–7 mm [56]); IDIFs extracted from cervical vessels are potentially less likely to be affected by spill-in and interindividual variability with respect to intracranial carotids [53] (Fig. 2). To further minimize PVE and spillover effects, aggressive voxel selection (e.g., via cluster analysis) [34, 53, 57] and calibration approaches [3] can be included. While IDIF calibration methods requiring one or more blood samples are expected to be the most accurate [58], PET/MRI allows for easier implementation of blood-free calibration, which relies on recovery coefficients estimated by knowing the carotid volume and scanner PSF [37]. One notable drawback of the current scenario is that IDIF validation studies in the brain have been performed mainly on healthy controls [59].

In sum, PET/MRI IDIF methods have thus been proposed for brain PET, especially for [^18^F]FDG [34, 37, 53, 60] and [^15^O]H_2_O [61, 62], with satisfactory results compared to the gold-standard AIF. Multiple automatic pipelines specifically designed for PET/MR have also been made publicly available, including fully automated vessel segmentation and sophisticated blood-free partial volume correction (PVC) [37, 60]. Overall, when a rigorous pipeline for input function extraction and correction is applied, IDIF approaches have been shown to match well with gold-standard AIF in terms of parameter estimates and test–retest reliability, not only in cardiac studies [17], but also in the brain [37], exceeding the performance of PBIF and SIME [59].

### IDIF application in the PET community: what is the “state of affairs”?

We sought to assess how the PET community perceives IDIF approaches, both in terms of their limitations and the efforts that have been made to improve their robustness and reproducibility. To this end, a survey was disseminated to several teams with a track record of quantitative PET imaging, through a) social media (LinkedIn), b) a list of emails compiled starting from a literature search on PubMed (keywords “image-derived input function” and “PET”). The authors of the identified papers were selected after excluding publications in animal models. A copy of the survey and the full survey results are reported in Supplementary Materials. When indicated by an asterisk (*), survey participants were allowed to give more than one answer to the question.

**Fig. 3:**
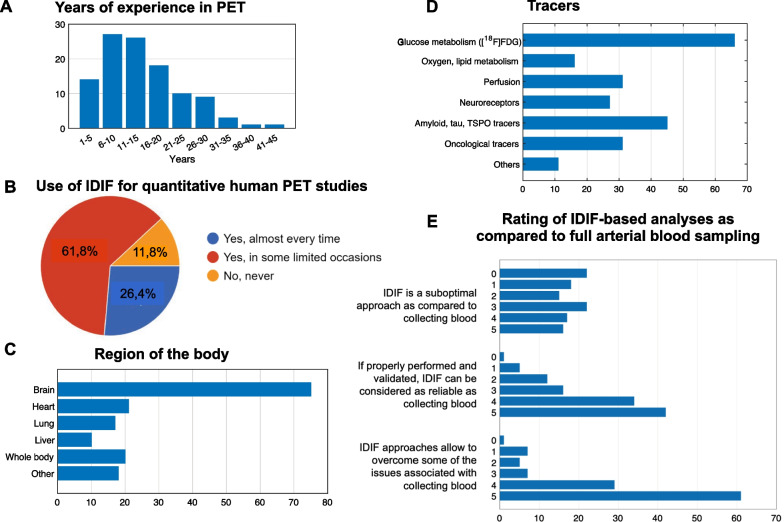
Survey results: participants. Information on survey respondents, with respect to years of experience in PET imaging (panel **A**), frequency of IDIF use in their PET studies (**B**), region of the body (**C**), and PET tracers (**D**) for which IDIF was applied, and overall opinion on IDIF approaches on a scale from 0 (complete disagreement) to 5 (complete agreement) (**E**). When not otherwise specified, the axes refer to the absolute number of answers

In total, 110 researchers responded to the survey (Fig. 3, Additional file 1: Figs. S1–S4), and most (88%) had used IDIF in their studies. Survey respondents indicated they had used IDIF approaches in quantitative PET studies comparing patient populations to healthy volunteers more frequently (68%) than in methodological studies with healthy volunteers only (Fig. 3B, Additional file 1: Figs. S5, S6). This likely reflects that the applicability of IDIF has increased, perhaps due to the aforementioned automated routines for cardiac and whole-body applications. In addition, respondents used IDIF most frequently for brain PET (75%), with smaller percentages for heart, lung, liver, and whole-body PET imaging*. Notably, 18% of survey respondents also reported applying IDIF to other organs, including kidneys, intestine, prostate, breast, muscle, bone, and adipose tissue (Fig. 3C, Additional file 1: Fig. S7).

The most commonly used PET tracers in IDIF studies* were [^18^F]FDG (*n* = 66), [^15^O]H_2_O (*n* = 31), amyloid (*n* = 21) and TSPO (*n* = 19), and various oncological tracers (*n* = 31) (Fig. 3D, Additional file 1: Fig. S8). Thus, many respondents had used IDIF approaches when radiometabolites had no significant impact on plasma tracer concentration ([^18^F]FDG, [^15^O]H_2_O).

With regard to PET scanners*, respondents indicated that they performed many IDIF studies on Siemens/CTI ECAT EXACT HR + (*n* = 28) and Siemens Biograph mMR (*n* = 30) scanners, but they also reported using GE Discovery MI PET/CT (*n* = 19), Siemens Biograph mCT (*n* = 18), GE Signa PET/MR (*n* = 17), Siemens Biograph Vision 600 (*n* = 13), Siemens Biograph Vision Quadra (*n* = 12), Siemens HRRT (*n* = 12), Philips Gemini TF 64 PET/CT (*n* = 11), and UIH uEXPLORER total-body scanner (*n* = 5) (Additional file 1: Fig. S9). While these results reflect in large part the local availability of each scanner system (e.g., the older HR + was prevalent in many centers), they also highlight how newer scanners are providing the opportunity for researchers to conduct more IDIF studies; salient examples include the Biograph mMR, which led to the development of multiple IDIF pipelines for brain PET, and the next-generation scanners (Quadra, uEXPLORER).

When asked to rate IDIF approaches versus arterial sampling (Fig. 3E, Additional file 1: Fig. S10), most survey respondents expressed positive views on the potential of IDIF to yield results comparable to AIF if properly validated. They further endorsed the possibility that IDIF might overcome some of the drawbacks of conventional arterial sampling.

**Fig. 4:**
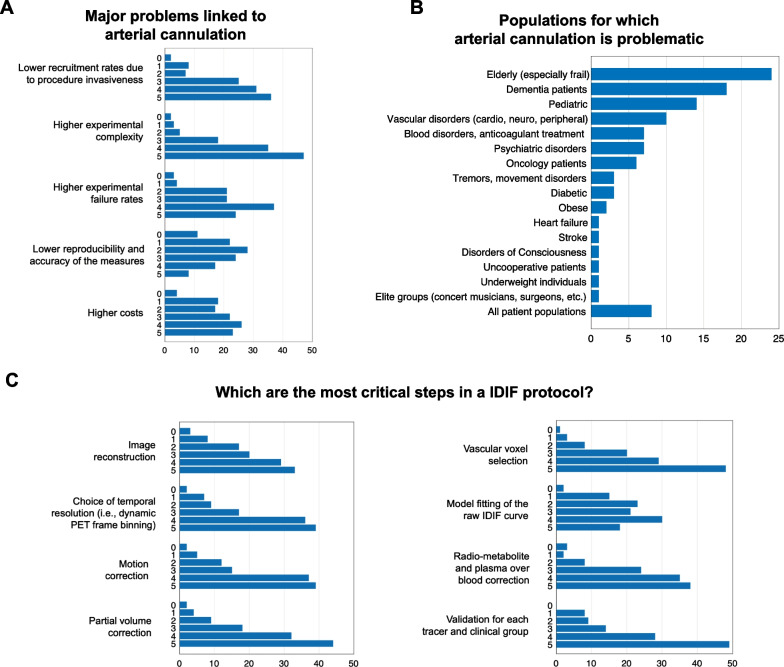
Survey results: arterial blood sampling and IDIF protocol. Arterial blood sampling—survey respondents were asked to rate how problematic a series of issues related to arterial sampling were, on a scale from 0 (not problematic) to 5 (very problematic) (panel **A**), and for which populations it was more difficult to conduct a full PET acquisition with arterial sampling (**B**). IDIF protocol—survey respondents rated how critical each of the common steps in an IDIF pipeline was (from image reconstruction to validation) on a scale from 0 (not critical) to 5 (very critical) (**C**). The *X*-axes refer to absolute number of answers

As to the most problematic issues for arterial sampling, the experimental complexity (Fig. 4, Additional file 1: Fig. S11), resulting in high failure rates and recruitment difficulties, was indicated; the higher costs and issues with reproducibility and accuracy were deemed less critical (Fig. 4A). Mixed assessments were reported regarding the risks of arterial cannulation to study participants (Additional file 1: Fig. S12). Among clinical populations, elderly participants, dementia patients, pediatric populations, and vascular disorders were deemed more problematic*, followed by psychiatric, hematological, and oncological disorders. Interestingly, while most survey respondents endorsed the view that the most problematic population is non-cooperative participants, some considered *all* patient populations as problematic (Fig. 4B, Additional file 1: Fig. S13).

When asked to comment on the importance of pre-processing and processing steps for IDIF (Fig. 4C, Additional file 1: Fig. S14), survey respondents endorsed PVC, voxel selection, and protocol validation as the most critical. They also unanimously selected the aorta as the best vascular site for IDIF extraction (60 participants gave it a rating of 5), followed by the cardiac chambers (Additional file 1: Fig. S15). This mirrors the scientific literature, including the pipelines for cardiac and whole-body applications described above [15]. Medium-sized arteries and venous vessels were perceived as more problematic, due to PVEs and different physiology, respectively.

As to imaging modalities for vessel segmentation, survey respondents selected early dynamic PET frames (most ratings = 4, 5) as the preferred method; notably, this method avoids coregistration. MR sequences, which are frequently acquired either sequentially or simultaneously, were also highly rated (Additional file 1: Fig. S16). Interestingly, when asked about voxel selection* (Additional file 1: Fig. S17), most survey respondents reported that they still perform manual delineation of vessels in MR or PET images (*n* = 54) and identify the highest activity voxels in the summed early PET frames (*n* = 53). However, more sophisticated approaches involving automatic vessel delineation and/or clustering of dynamic PET data, are also employed by many survey respondents (*n* = 24 and *n* = 28, respectively), reflecting the broader availability of and greater interest in automated pipelines.

With regard to kinetic estimates obtained with IDIF approaches (Additional file 1: Fig. S18), respondents deemed that tissue-to-blood ratios and macroparameters like *K*_i_ or *V*_T_ from graphical methods were reliable (more than 50 survey respondents gave this item a rating of 4). Opinion was more mixed for microparameters derived from full-compartment modeling, which require a more accurate description of full IDIF kinetics.

### Next-generation PET scanners and the new vision for IDIF

In recent years, PET scanner sensitivity and spatial resolution have improved, with longer axial FOVs (i.e., LAFOV), leading to impressive advancements in whole-body imaging (total-body scanners, e.g., uEXPLORER, < 3 mm FWHM, axial FOV 194 cm [63]; Vision Quadra, 3.3 FWHM, axial FOV 106 cm [64]), as well as organ-dedicated imaging (e.g., NeuroEXPLORER, < 1.8 mm FWHM, axial FOV 48 cm [65]; Prism-PET, 1 mm FWHM, axial FOV 25.5 cm [66], ultra-high resolution PET, 1.3 mm FWHM, axial FOV 27.1 cm [67]).

The most relevant improvements followed the development of solid-state PET detectors—which have had a remarkable impact on PET sensitivity (> 20 counts per second/kBq)—as well as improvements in spatial resolution (< 3 mm) and temporal resolution, especially due to depth-of-interaction capabilities and time-of-flight (TOF) information, with time resolution reaching 200 picoseconds [16]. These remarkable advances have led to a renaissance in both quantitative PET research [14, 29] and IDIF specifically.

**Fig. 5:**
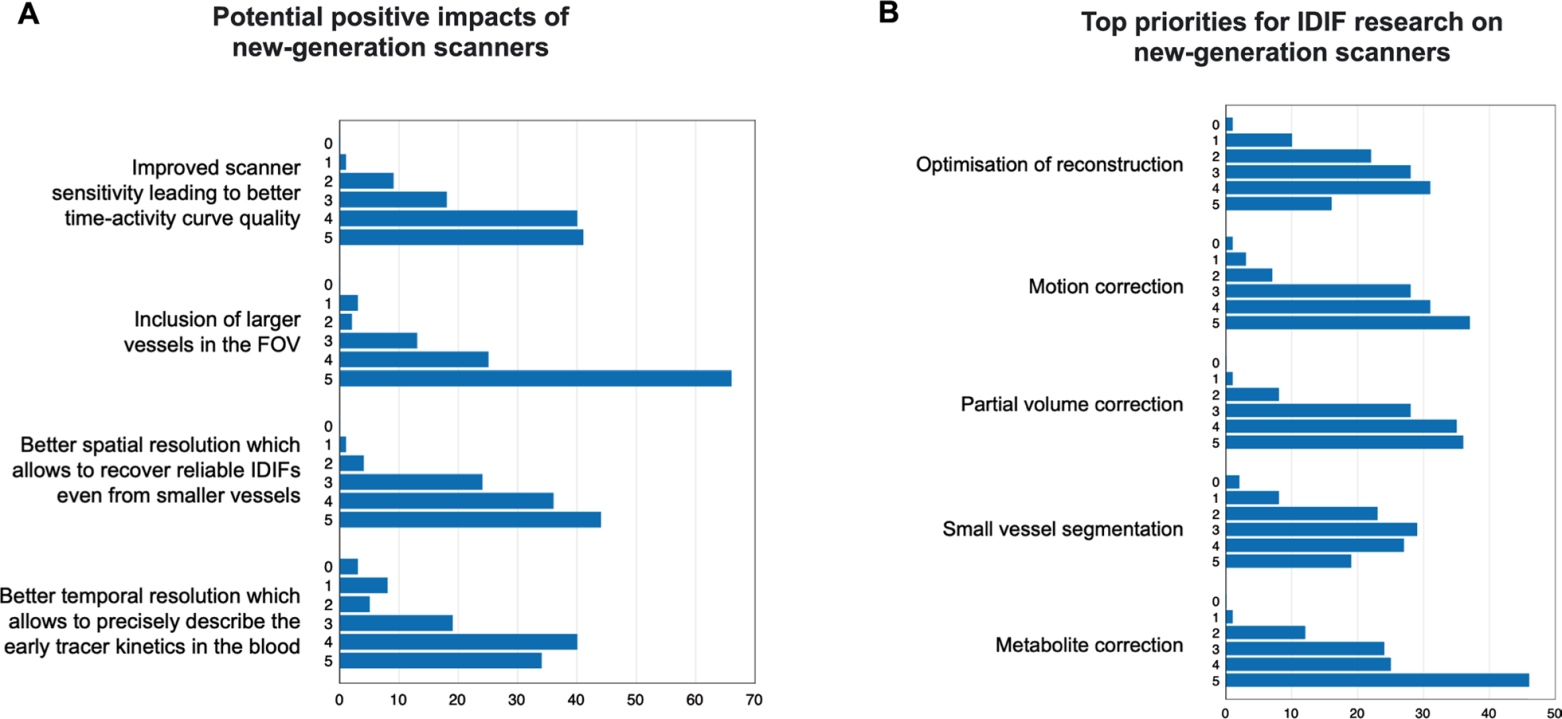
Survey results: IDIF and next-generation PET scanners. The participants were asked to rate the potential impacts that new scanners may have on IDIF research (**A**) and the top priorities for IDIF research with next-generation scanners (**B**) on a scale from 0 (no importance) to 5 (high importance). The *X*-axes refer to absolute number of answers

When exploring the PET community’s perception, the greatest advantage of next-generation scanners was considered to be the inclusion of large vessels in the FOV, particularly the aorta (Fig. 5, Additional file 1: Fig. S19); survey respondents also endorsed improved scanner sensitivity, more precise description of the early tracer kinetics, and improved spatial and temporal resolution, which allow more reliable IDIF extraction from smaller vessels. Another perceived advantage of whole-body and total-body PET is the possibility of obtaining metabolite information from multi-organ kinetic modeling (Additional file 1: Fig. S21) (see Sect. 4.3).

When asked about the most pressing issues to address with these new capabilities, survey respondents considered metabolite correction to be the main challenge. Motion correction and PVC were also considered critical problems, followed by optimization of reconstruction and small vessel segmentation approaches (Additional file 1: Fig. S20).

We will now discuss more in depth these potential advantages of next-generation scanners and how they may make IDIF approaches more robust and more widely applicable.

#### A game changer for large and small vessels

New scanners may effectively minimize the problem of PVEs by either imaging large vessels thanks to a longer FOV, or by imaging smaller vessels with a higher spatial resolution and sensitivity.

**Fig. 6:**
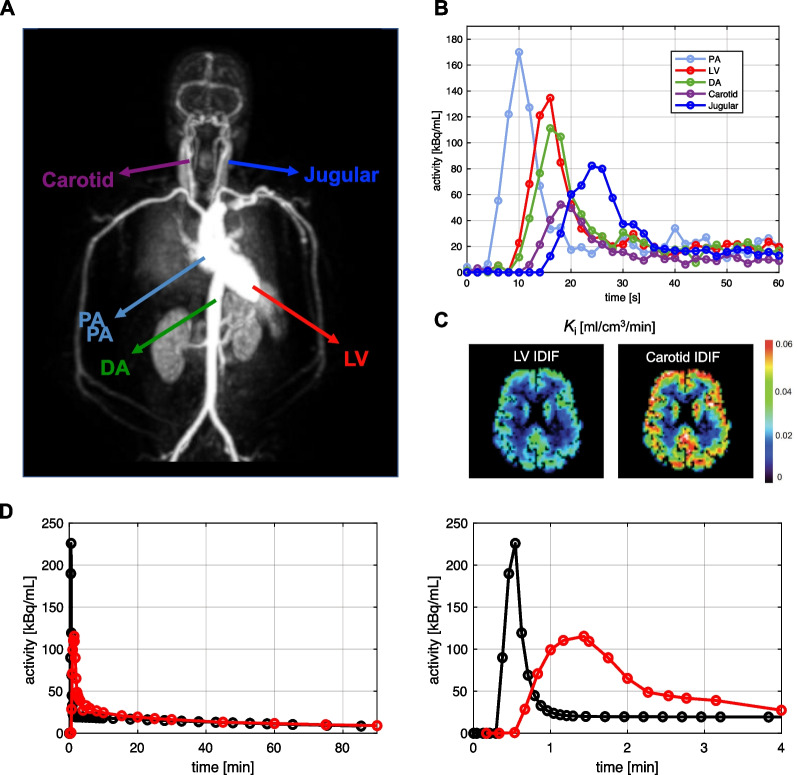
Comparing delay and dispersion of multiple IDIF sites. Impact of IDIF extraction site on input delay/dispersion and kinetic parameter estimates, specifically [^18^F]FDG *K*_i_ in the brain. Total-body PET maximum intensity projection image of different vascular sites for IDIF extraction, i.e., pulmonary artery (PA, ~ 3 cm), left ventricle (LV, ~ 4 to 6 cm), descending aorta (DA, ~ 2.5 to 3 cm), common carotid arteries (~ 7 mm), jugular veins (~ 0.8 to 1.2 cm) (**A**). Simulated IDIF curves representing the delay/dispersion characteristics of each site (**B**). *K*_i_ parametric maps (brain), showing how *K*_i_ is higher for the carotid IDIF due to the smaller IDIF AUC with respect to the LV (**C**). Differences in delay and dispersion between IDIF extracted from the aorta (black) and AIF obtained from radial artery (red) in a simulated [^18^F]FDG PET study; the full-time course is shown on the left, and the first four minutes on the right (**D**)

The growing availability of LAFOV scanners (especially total-body scanners) has become easier to include large blood pools such as the ascending and descending aorta [4, 49, 68], or the left ventricle [69], in the FOV, which has fueled a renewed enthusiasm for IDIF approaches, both for the brain and other organs. The presence of large vessels has markedly simplified the steps needed for a robust IDIF extraction pipeline because vessel segmentation and voxel selection have become much easier (see “Main text” Section) [15, 70]. Motion artifacts (especially those related to respiration) significantly impact thoracic PET acquisitions [16, 29], potentially resulting in severe misplacement of the ROIs positioned for IDIF extraction [51]. However, the choice of the aorta seems a good compromise when considering motion and PVEs [68]. Moreover, advanced spatiotemporal reconstruction algorithms (which go beyond independent reconstruction of each PET frame) are beginning to take respiratory motion into account, thus facilitating IDIF extraction from thoracic vessels [71]. Another aspect that needs to be taken into account is the fact that the activity measurements in large arteries and heart exhibit an earlier, higher and narrower peak than those from peripheral arteries [4]. Peripheral arteries are closer to the activity in arterioles and capillaries (i.e., the local input to the tissue system) in terms of delay (i.e., variable appearance time of radioactivity in the blood in different sites depending on distance and blood velocity) and dispersion (i.e., the smearing of the blood radioactivity curve due to inhomogeneity in blood velocity fields, causing a change in the shape of the input) (Fig. 6).

Delay and dispersion have little impact on parameter estimation approaches based on the IDIF area under the curve (AUC), like graphical methods [4]; on the other hand, if microparameters are of interest, especially *K*_1_ which is a scaling factor of the input function, more attention to the shape of the whole IDIF curve is required. Adjustments for delay and dispersion thus need to be taken into account if comparing the aorta IDIF with a reference AIF drawn from the radial artery (Fig. 6D) [4, 37, 59, 72]. Incorporating IDIF time delays for different organs [72] and addressing the effect of dispersion [73] via joint estimation were both shown to have significant impact on kinetic estimates in total-body PET. A further development could be to use deep learning for predicting delay and dispersion along with the input function at the voxel level [74].

Next-generation scanners are also expected to improve imaging of smaller vessels, like the carotids (~ 4 to 7 mm) and femoral arteries (~ 8 mm). Spillover and PVEs, in particular, should be considerably reduced in response to the high sensitivity and spatial resolution of these systems, which allow reliable selection of vascular voxels without risk of contamination from surrounding tissues. Nonetheless, even in PET studies with high-performance scanners (Vision Quadra, PennPET Explorer, uEXPLORER), IDIF extraction from the carotid arteries has not been considered reliable when simple ROI placement is used [68–70], unless more sophisticated PVC is implemented [75].

In contrast, brain-dedicated PET scanners such as the NeuroEXPLORER are expected to perform markedly better; as an example, such scanners are able to image the internal carotids with unprecedented spatial resolution (< 2 mm). An axial FOV of ~ 50 cm also makes it possible to extend the assessment to the neck vessels in their entirety, obtaining a more precise estimate of the activity in the common carotids, as well as information from the aortic arch for comparison [65].

#### High temporal resolution and accurate IDIF sampling

**Fig. 7:**
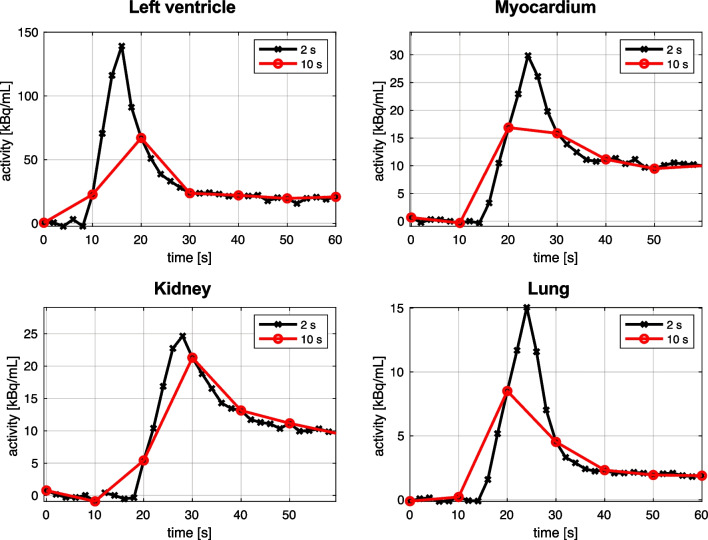
Impact of temporal resolution on PET tracer kinetics. Assessing the impact of higher (two-second frames, in black) and lower temporal resolution (10-s frames, in red) on regional time-activity curves in different regions of interest: left ventricle, myocardium, kidney, and lung. Simulation reproducing the first minute of data from a dynamic [^18^F]FDG PET scan performed on a scanner with ultra-high temporal resolution

The exceptional sensitivity of new PET systems, in conjunction with dedicated spatiotemporal reconstruction algorithms [76], allows to reconstruct frames with a higher number of counts: This leads to the possibility to achieve high temporal resolution, with 1–2 s or even sub-second framing (Fig. 7). Following how IDIF kinetics change over time, and accurately estimating the IDIF peak, can be important for specific parameters (i.e., *K*_1_, which is sensitive to the early input function); however, for tracers with high metabolite fraction (e.g., [^11^C]PBR28 [3], [*carbonyl*-^11^C]WAY-100635 [25]), the marked drop in the tail AUC after metabolite correction makes the accurate description of the peak important for other parameters as well (e.g., *V*_T_).

While IDIFs from next-generation scanners have been mainly tested with simplified quantification approaches [4, 68] that only rely on the input AUC, reliable estimation of the peak will make more complex modeling approaches more feasible, particularly the ability to estimate microparameters via compartmental modeling [14, 70].

An additional perspective offered by high-temporal-resolution PET imaging is the ability to expand the range of applicability of conventional kinetic modeling. For instance, a standard one-tissue compartment model describing early [^18^F]FDG PET data (0–3 min, binned into 2 s-frames, uEXPLORER scanner, fast [^18^F]FDG bolus) was outperformed by a *time-varying* kinetic model which allowed to resolve a vascular phase (blood flow) and tissue uptake phase (tracer delivery *K*_1_) in a single scan [14, 77].

#### Can multi-organ kinetics provide information for metabolite correction?

Respondents in our survey indicated that radiometabolite correction as the most difficult obstacle to overcome for widespread IDIF implementation. While considerable skepticism exists regarding whether next-generation scanners can address this issue, it is intriguing to consider the possibility that total-body imaging systems such as the uEXPLORER (or whole-body acquisitions on clinical scanners) might allow the development of whole-body physiological models of radiotracer metabolism, thus enabling accurate estimation of the parent input function without needing metabolite information obtained via arterial sampling [14]. While whole-body physiologically based pharmacokinetic models have been extensively employed in drug development [78], no well-validated data on their application to PET kinetic modeling exist yet. Ideally, once the appropriate compartment model for a given tracer’s parent fraction is formulated [79], whole-body PET data (from liver, lung, spleen etc.) would provide information that could be used to estimate the model parameters [80]. Deep learning approaches could potentially be used to make these highly nonlinear whole-body models more efficient to estimate and use [74].

Regardless, richer PET data from a spatial and temporal perspective might provide additional power to approaches that have already shown some promise, such as combining IDIF with SIME-based approaches to obtain a metabolite-corrected input function [59, 81].

## Conclusions

Significant improvements in PET scanner technology achieved in the last decade, such as high sensitivity, high spatiotemporal resolution, and long axial FOV, may effectively solve some of the limitations associated with IDIF, such as voxel-level noise, partial volume effects and poor temporal sampling, making IDIF approaches more feasible and reliable. These advances, along with more reliable automated IDIF extraction approaches, may allow reliable quantification of the activity concentrations in the blood, including during the early peak, a necessary prerequisite for quantification with compartmental modeling. This leads to the intriguing possibility of potentially obtaining not only unbiased and precise macrokinetic parameters, but also microparameters (e.g., *K*_1_ [19]), thus entering into a new era of exploration for physiology and pathophysiology [82–84], and finally reaching clinical application of absolute quantification of PET data. With these perspectives, exciting times lie ahead.

Nevertheless, individualized radiometabolite correction remains an unsolved challenge. A theoretical approach, for which no viable demonstration exists yet, would be to estimate tracer metabolism from multiple organ kinetics in a dynamic whole-body scan. Until this issue can be solved effectively, IDIF may continue to be marginally employed both in clinics and in research, with the exception of a few tracers ([^18^F]FDG).

### Supplementary Information

**Additional file 1**. Supplementary file.

## Data Availability

All data generated or analyzed during this study are included in this published article (and its supplementary information files).

## Abbreviations

AIF: Arterial input function
AUC: Area under the curve
CT: Computed tomography
FBP: Filtered back-projection
FWHM: Full width at half maximum
[^18^F]FDG: [^18^F]fluorodeoxyglucose
FOV: Field of view
IDIF: Image-derived input function
LAFOV: Long axial field of view
MBF: Myocardial blood flow
MRI: Magnetic resonance imaging
OSEM: Ordered subset expectation maximization
PBIF: Population-based input function
PET: Positron emission tomography
POB: Plasma-over-blood ratio
PSF: Point spread function
PVE: Partial volume effect
PVC: Partial volume correction
ROI: Region of interest
SIME: Simultaneous estimation
SNR: Signal-to-noise ratio
TAC: Time-activity curve
TOF: Time-of-flight
