# Supplementary material for: An update on the use of image-derived input functions for human PET studies: new hopes or old illusions?
Source: EJNMMI Res. 2023 Nov 10;13:97. doi: 10.1186/s13550-023-01050-w (PMC10638226; doi:10.1186/s13550-023-01050-w)
Supplement: Supplementary file 1 — Additional file 1. Supplementary file. [file 13550_2023_1050_MOESM1_ESM.docx]

**Supplementary Materials**

**Survey**

**An update on the use of image-derived input functions for human PET imaging studies: new hopes or old illusions?**

**Background**

The need for arterial blood sampling in quantitative human PET studies is one of the major factors limiting a wider usability of this imaging method in research and clinical settings.

Non-invasive approaches based on image-derived signals, i.e., image-derived input function (IDIF), have been proposed as possible alternatives, and have been applied especially in cardiac and whole-body dynamic imaging. However, this solution has been faced with contrasting feelings and mixed results, especially for brain PET, where molecular imaging studies using IDIF represent a minority.

Recently, the introduction of new-generation PET scanners with higher sensitivity, superior spatial and temporal resolution, and longer axial field of view (LAFOV) allowing inclusion of large vessels like the aorta, has brought IDIF methods back into the spotlight, as the features of these state-of-the-art scanners have the potential to address several key limitations of IDIF.

**Rationale of the survey:**

- To gather information on the opinions and experience of the PET community with IDIF approaches in human studies
- To set the scene for the implementation of IDIF protocols with new-generation PET scanners

***About the rater***

- **Background**
  - 1. Physicist
    2. Biomedical Engineer
    3. Physician
    4. Pharmacist
    5. Chemist
    6. Biologist
    7. Other
- **Years of experience in PET imaging**
- **Country (state) of current main employment**
- **Main employment type**
  - 1. Academia
    2. Industry
- **Have you ever used IDIF for quantitative human PET studies?**
  - 1. Yes, almost every time
    2. Yes, in some limited occasions
    3. No, never
- **If so, did you apply IDIF to quantitative PET studies comparing patient populations to healthy controls?**
  - 1. Yes
    2. No, I have only applied it to healthy subjects for methodological purposes
- **For which region in the body have you used an IDIF approach?**
  - 1. Brain
    2. Heart
    3. Lung
    4. Liver
    5. Whole body
    6. Other

**In case you chose "other", please specify the body district(s) you have applied IDIF to.**

- **For which PET tracers have you used an IDIF approach?**
  - 1. [18F]fluorodeoxyglucose (glucose metabolism)
    2. [15O]water (blood flow)
    3. [11C]UCB-J (synaptic density)
    4. [11C]PK11195, [11C]PBR28, [11C]ER176, [18F]DPA714 (microglial activation)
    5. [11C]PiB, [18F]flutemetamol, [18F]florbetapir, [18F]florbetaben (amyloid deposition)
    6. [11C]raclopride, [11C]-(+)-PHNO (dopamine receptors)
    7. [carbonyl-11C]WAY-100635, [18F]altanserin (serotonin receptors)
    8. [11C]flumazenil, [11C]Ro15-4513 (GABA receptors)
    9. [11C]ABP688, [18F]GE179 (glutamate receptors)
    10. Other

**In case you chose "other", please specify the PET tracer(s) you have applied IDIF to.**

- **On which PET scanners have you performed studies with an IDIF approach?**
  - 1. Siemens/CTI ECAT EXACT HR+
    2. Siemens HRRT
    3. Siemens Biograph mMR
    4. GE Discovery MI PET/CT
    5. GE Signa PET/MR
    6. Philips Gemini TF 64 PET/CT
    7. Siemens Biograph mCT
    8. Siemens Biograph Vision 600
    9. Siemens Biograph Vision Quadra
    10. EXPLORER total-body scanner
    11. Other

**In case you chose "other", please specify the PET scanner(s) you have worked on.**

- **If you had to read or review a paper using IDIF to generate blood input functions, how would you consider the work compared to full arterial blood sampling and analysis? Rate each statement on a scale from 0 (do not agree) to 5 (agree completely).**
  - 1. IDIF is a suboptimal approach as compared to collecting blood
    2. If properly performed and validated, IDIF can be considered as reliable as collecting blood
    3. IDIF approaches allow to overcome some of the issues associated with collecting blood

***Part 1 – The problems of the arterial line***

- **Which do you think are the most problematic issues when dealing with an arterial line in a PET imaging experiment? Rate each option on a scale from 0 (not problematic) to 5 (very problematic).**
  - 1. Lower recruitment rates due to procedure invasiveness
    2. Higher experimental complexity
    3. Higher experimental failure rates
    4. Lower reproducibility and accuracy of the measures
    5. Higher costs
- **How risky do you consider arterial cannulation for study participants? Rate it on a scale from 0 (not risky) to 5 (very risky).**
- **For which clinical populations do you think this procedure is most problematic?**

***Part 2 – IDIF application***

- **How critical do you consider each of the following steps in an IDIF protocol? Rate each option on a scale from 0 (not critical) to 5 (very critical).**
  - 1. Image reconstruction
    2. Choice of temporal resolution (i.e., dynamic PET frame binning)
    3. Motion correction
    4. Partial volume correction
    5. Vascular voxel selection
    6. Model fitting of the raw IDIF curve
    7. Radio-metabolite and plasma over blood correction
    8. Validation for each tracer and clinical group
- **Which cardiovascular structures do you think are most suitable for IDIF extraction? Rate each option on a scale from 0 (not suitable) to 5 (very suitable).**
  - 1. Cardiac chambers (ventricles, atria)
    2. Aorta
    3. Medium caliber arteries (carotid, femoral, renal etc.)
    4. Large venous vessels (vena cava, jugular etc.)
- **When vessel segmentation is used in IDIF extraction protocols, which imaging modalities do you think are most suitable? Rate each option on a scale from 0 (not suitable) to 5 (very suitable).**
  - 1. MRI (time-of-flight MR angiography, T1w etc.)
    2. CT
    3. Dynamic PET (early frames)
- **In your experience with IDIF, how did you select the voxels for deriving the raw IDIF time-activity curve?**
  - 1. Clustering on full dynamic PET images
    2. Highest activity voxels in summed PET image of the initial frames
    3. Automatic segmentation on MR images
    4. Manual delineation on MR or PET images
    5. Other

**In case you chose "other", please specify the voxel selection approach you used.**

- **How reliable do you consider the following parameters when obtained with an IDIF approach?  Rate each option on a scale from 0 (not reliable) to 5 (very reliable)**
  - 1. Tissue-to-blood ratios
    2. Macro-parameters from Logan or Patlak’s graphical methods
    3. Micro-parameters/single kinetic rates from compartmental modeling

***Part 3 – New-generation PET scanners***

- **Which could be the positive impacts of new-generation scanners on IDIF extraction? Rate each option on a scale from 0 (no impact) to 5 (high impact).**
  - 1. Improved scanner sensitivity leading to better time-activity curve quality
    2. Inclusion of larger vessels in the FOV
    3. Better spatial resolution which allows to recover reliable IDIFs even from smaller vessels
    4. Better temporal resolution which allows to precisely describe the early tracer kinetics in the blood
- **Which do you think are the top-priority issues in IDIF research which should be addressed with the help of next-generation PET scanners? Rate each option on a scale from 0 (low priority) to 5 (high priority).**
  - 1. Optimization of reconstruction
    2. Motion correction
    3. Partial volume correction
    4. Small vessel segmentation
    5. Metabolite correction
- **A key potential of whole-body and total-body PET acquisitions with next-generation scanners is believed to be the possibility of obtaining metabolite information from kinetic modelling  of multi-organ data. Rate how promising you think such approach could be for metabolite correction of IDIF on a scale from 0 (not promising) to 5 (very promising).**

***Addendum***

- **For each of the following metabolite correction approaches, please provide a list of PET tracers to which the chosen correction can reliably be applied.**

1. **No correction**
2. **Correction through a population metabolite curve**
3. **Correction through a population metabolite curve rescaled with a single blood sample**

**4. Correction through an individual metabolite curve obtained from venous samples.**

E.g., 1. Tracer A, Tracer B ...

        2. Tracer X, Tracer Y ...

        3. ....

        4. ....

- **If you have any additional comments or suggestions related to these questions, please provide them here.**

**Full survey results**

**Note**: When not otherwise specified, the figure axes display the absolute number of given answers.

***About the survey respondents***

- **Background** (*Supplementary Figure S1*)

- **Years of experience in PET imaging** (*Supplementary Figure S2*)

- **Country (state) of current main employment** (*Supplementary Figure S3*)

- **Main employment type** (*Supplementary Figure S4*)


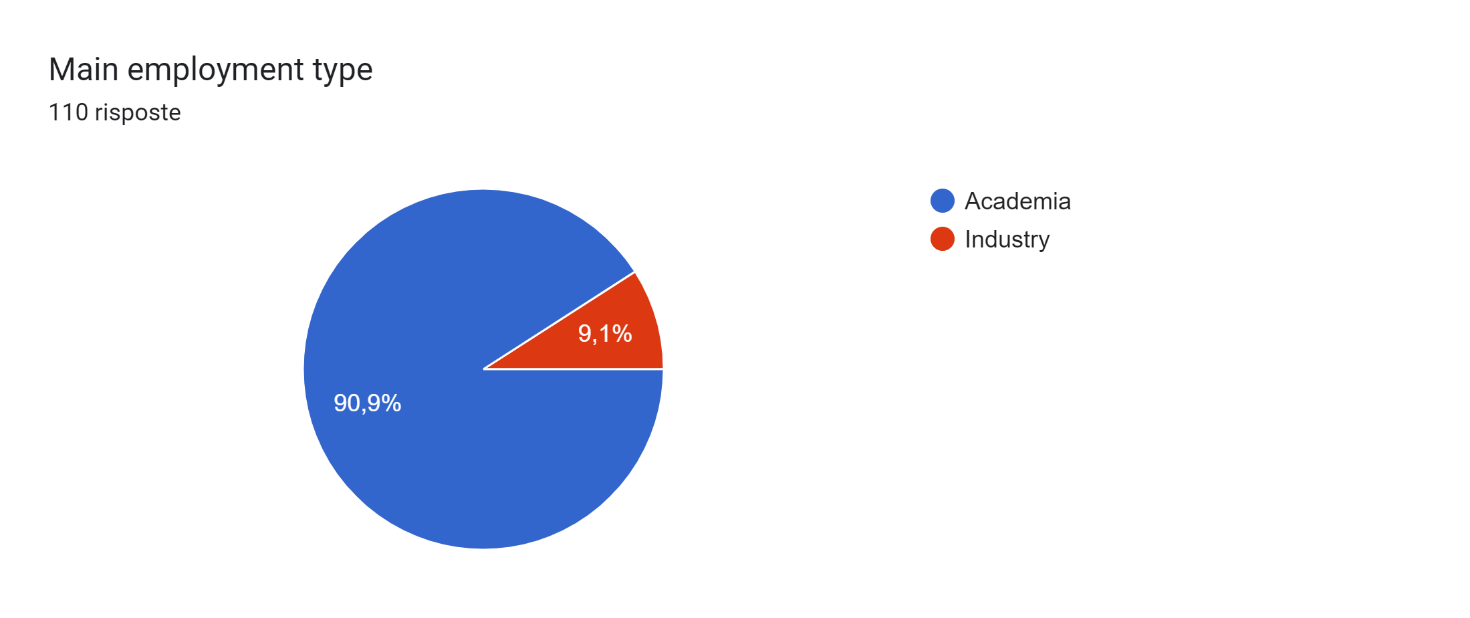


- **Have you ever used IDIF for quantitative human PET studies?** (*Supplementary Figure S5*)

**
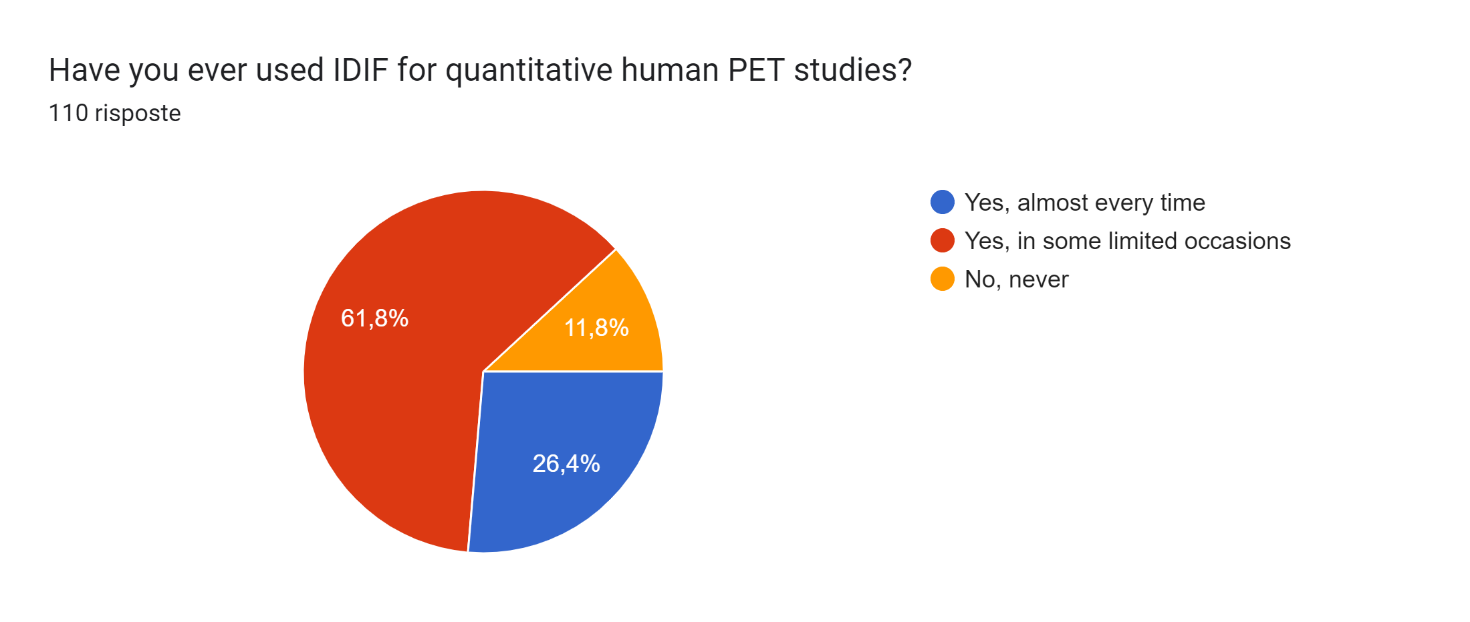
**

- **
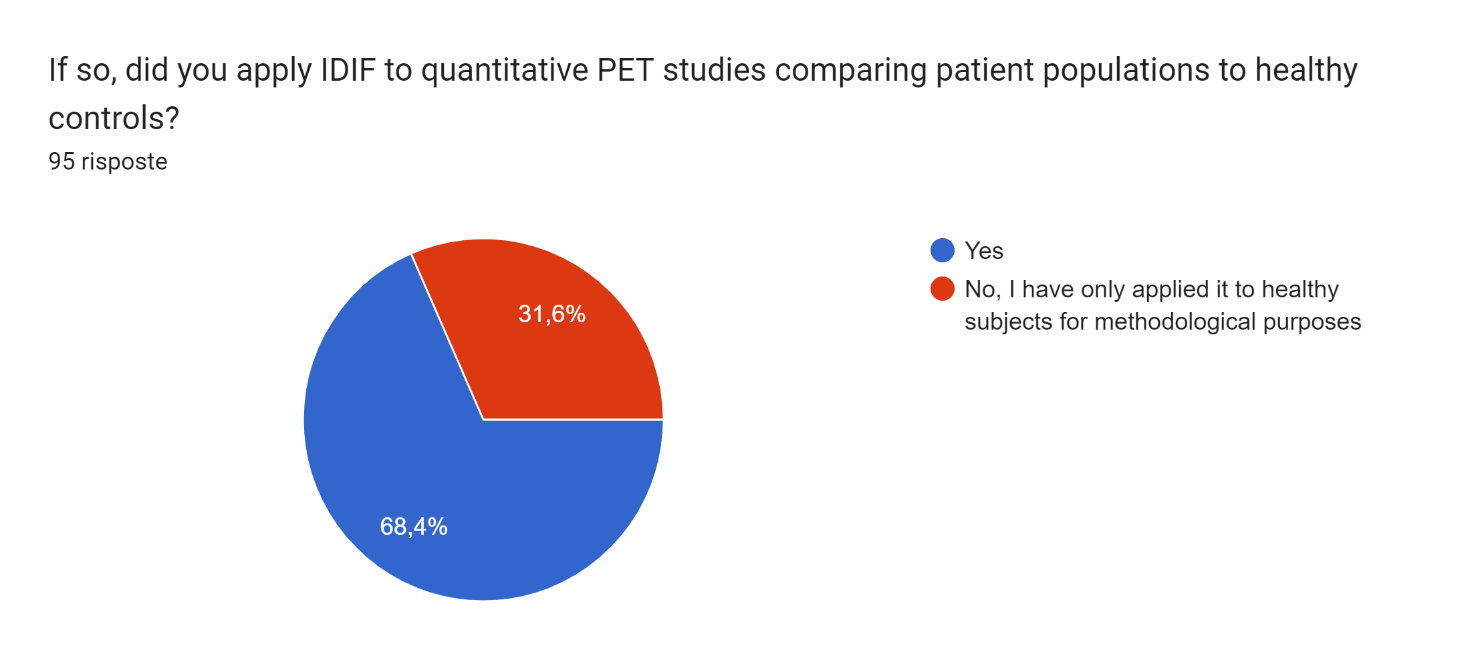
If so, did you apply IDIF to quantitative PET studies comparing patient populations to healthy controls?** (*Supplementary Figure S6*)
- **For which region in the body have you used an IDIF approach?** (*Supplementary Figure S7*)

**In case you chose "other", please specify the body district(s) you have applied IDIF to.**

Kidneys

Pelvic organs

Intestine

Prostate

Breast

Muscles

Bone

Adipose tissue

- **For which PET tracers have you used an IDIF approach?** (*Supplementary Figure S8*)

**Legend:**

**Glucose metabolism:** [^18^F]FDG

**Oxygen metabolism:** [^15^O]O2

**Lipid metabolism:** [^11^C]palmitate, [^18^F]FTHA, [^11^C]arachidonic acid, [^11^C]acetate, [^11^C]acetoacetate

**Perfusion:** [^15^O]water, [^11^C]butanol

**Blood volume:** [^15^O]CO

**SV2A tracers:** [^11^C]UCB-J, [^18^F]SynVesT-1

**Dopamine** **system**: [^11^C]raclopride, [^11^C]-(+)-PHNO, [^18^F]FE-PE2I, [^18^F]FDOPA

**Serotonin system:** [carbonyl-^11^C]WAY-100635, [^18^F]altanserin

**GABA system**: [^11^C]flumazenil, [^11^C]Ro15-4513

**Glutamate system:** [^11^C]ABP688, [^18^F]GE179

**Other neurotransmitter systems:** [^11^C]MK-8278 (histamine receptors) [^11^C]TMSX, [^18^F]CPFPX, [^11^C]HED

**TSPO tracers:** [^11^C]PK11195, [^11^C]PBR28, [^11^C]ER176, [^18^F]DPA714

**Amyloid tracers**: [^11^C]PiB, [^18^F]flutemetamol, [^18^F]florbetapir, [^18^F]florbetaben

**Tau tracers:** [^18^F]MK-6240, [^18^F]AV-1451, [^18^F]flortaucipir, [^18^F]PI-2620

**Bone tumors:** [^18^F]NaF, [^18^F]fluoride

**Hypoxia** **tracers:** [^18^F]FMISO, [^18^F]FAZA

**PSMA tracers:** [^18^F]flucyclovine

**Tumor metabolism:** [^11^C]choline, [^18^F]FMC, [^11^C]-L-Methionine

**Tumor proliferation:** [^18^F]FET, [^18^F]FLT

**Other** **oncological tracers:** [^68^Ga]Ga-DOTA-TOC, [^89^Zr]crefmirlimab, integrin αvβ4 tracers

**Other:** [^18^F]AS2471907, [^11^C]glyburide, [^11^C]rolipram, [^11^C]MeDAS

- **On which PET scanners have you performed studies with an IDIF approach?** (*Supplementary Figure S9*)

**In case you chose "other", please specify the PET scanner(s) you have worked on.**

GE Discovery VCT PET/CT

Siemens Biograph 16

Siemens Biograph 40

Philips Vereos

Siemens BrainPET

GE Discovery 690 PET/CT

GE Advance

Philips Gemini 64

Siemens CTI ECAT 931

Shimadzu SET-2400W

GE DISCOVERY 610

- **If you had to read or review a paper using IDIF to generate blood input functions, how would you consider the work compared to full arterial blood sampling and analysis? Rate each statement on a scale from 0 (do not agree) to 5 (agree completely).** (*Supplementary Figure S10*)

***Part 1 – The problems of the arterial line***

- **Which do you think are the most problematic issues when dealing with an arterial line in a PET imaging experiment? Rate each option on a scale from 0 (not problematic) to 5 (very problematic).** (*Supplementary Figure S11*)

- **How risky do you consider arterial cannulation for study participants? Rate it on a scale from 0 (not risky) to 5 (very risky).**(*Supplementary Figure S12)*

- **For which clinical populations do you think this procedure is most problematic?** (*Supplementary Figure S13*)

***Part 2 – IDIF application***

- **How critical do you consider each of the following steps in an IDIF protocol? Rate each option on a scale from 0 (not critical) to 5 (very critical).** (*Supplementary Figure S14*)

- **Which cardiovascular structures do you think are most suitable for IDIF extraction? Rate each option on a scale from 0 (not suitable) to 5 (very suitable).** (*Supplementary Figure S15*)

- **When vessel segmentation is used in IDIF extraction protocols, which imaging modalities do you think are most suitable? Rate each option on a scale from 0 (not suitable) to 5 (very suitable).** (*Supplementary Figure S16*)

- **In your experience with IDIF, how did you select the voxels for deriving the raw IDIF time-activity curve?** (*Supplementary Figure S17*)

**In case you chose "other", please specify the voxel selection approach you used.**

Automatic segmentation of blood vessels on CT images

Independent component analysis on PET images

Parametric clustering on PET voxel dynamics

- **How reliable do you consider the following parameters when obtained with an IDIF approach?  Rate each option on a scale from 0 (not reliable) to 5 (very reliable)** (*Supplementary Figure S18*)

***Part 3 – New-generation PET scanners***

- **Which could be the positive impacts of new-generation scanners on IDIF extraction? Rate each option on a scale from 0 (no impact) to 5 (high impact).** (*Supplementary Figure S19*)

- **Which do you think are the top-priority issues in IDIF research which should be addressed with the help of next-generation PET scanners? Rate each option on a scale from 0 (low priority) to 5 (high priority).** (*Supplementary Figure S20*)

- **A key potential of whole-body and total-body PET acquisitions with next-generation scanners is believed to be the possibility of obtaining metabolite information from kinetic modelling  of multi-organ data. Rate how promising you think such approach could be for metabolite correction of IDIF on a scale from 0 (not promising) to 5 (very promising).**(*Supplementary Figure S21*)

***Addendum***

- **For each of the following metabolite correction approaches, please provide a list of PET tracers to which the chosen correction can reliably be applied.**

1. **No correction**

[18F]FDG (36), [15O]Water (11), [18F]NaF (3), [68Ga]PSMA-11 (2), [18F]FET (1), [15O]CO (1), [11C]glyburide (1), [11C]erlotinib (1), [18F]FMISO (1), [11C]AZ10419369 (1), [18F]Fluoride (1), [11C]PK11195 (1), [11C]raclopride (1), 82Rb (1), [13N]NH_3_ (1), any 68Ga-labelled peptide that does not metabolise.

1. **Correction through a population metabolite curve**

None (7), [18F]FDG (2), [11C]SL25.1188 (1), [11C]acetate (1), [11C]palmitate (1), [11C]HED (1), [11C]metoclopramide (1), [18F]FLT (1), [18F]FMISO (1), [11C]UCB-J (1), [11C]CUMI101 (1), [15O]O2 (1), [11C]PK11195 (1), [18F]FDOPA - after carbidopa/entacapone administration (1)

1. **Correction through a population metabolite curve rescaled with a single blood sample**

None (7), [18F]FDG (2), [18F]FLT(2), [11C]Rolipram (1), [11C]PK11195 (1), [68Ga]Ga-DOTA-TOC (1), [18F]FMISO (1), [18F]GE179 (1), [18F]SynVesT-1 (1), [18F]FEPPA (1), [18F]FDOPA (1)

**4. Correction through an individual metabolite curve obtained from venous samples.**

None (3), [18F]FDG (1), [18F]FLT (1), [18F]FMISO (1), [11C]CUMI101 (1), [11C]DASB (1), [18F]FPEB (1), [11C]PBR28 (1), [11C]HED (1), [18F]GE-179 (1), [15O]tracers (1)

*Note: number of times a tracer was cited reported in parentheses.*

- **If you have any additional comments or suggestions related to these questions, please provide them here.**

New scanners can obviate the problem of PVE because larger vessels will be in the FOV, but even with new scanners the problem of radiometabolites remains.

IDIF is not the only less-invasive approach to quantification of PET data that would benefit from improved PET scanners (e.g., simultaneous estimation). SIME methods have the potential to avoid common errors such as PVEs.

The IDIF method has an extreme potential in obtaining curves with a better kinetic behavior in comparison to the AIF, because it is extracted from images, from which the tissue curves come as well.

The use of simplified approaches alternative to arterial sampling necessarily introduces a degree of error, which is acceptable in case of studies with large effect sizes, but this makes it difficult to extend these approaches to large/multi-site studies due to their lower reliability.

Many of the methodological questions in the survey are mainly relevant for brain imaging, while for cardiac imaging, reconstructions, PVE etc. are much less important because larger vessels are included in the FOV.

The questions are quite wide and sometimes tricky to answer based on the options provided.

Some of the questions above are a bit difficult to answer in a general way, as some of the statements are tracer specific, e.g. true for one tracer, but false for another.

Some of the answers depend a lot on target region and FOV. For instance, an AIF is probably not very useful in the lung (dispersion/delay), liver (dual input).
